# Ailanthus Altissima-derived Ailanthone enhances Gastric Cancer Cell Apoptosis by Inducing the Repression of Base Excision Repair by Downregulating p23 Expression

**DOI:** 10.7150/ijbs.60674

**Published:** 2021-07-05

**Authors:** Chun-ming Wang, Hua-fu Li, Xiao-kun Wang, Wu-guo Li, Qiao Su, Xing Xiao, Teng-fei Hao, Wei Chen, Ya-wei Zhang, Hai-yong Zhang, Wang Wu, Zhen-ran Hu, Guang-yin Zhao, Ming-yu Huo, Yu-long He, Chang-hua Zhang

**Affiliations:** 1 Digestive Disease Center, The Seventh Affiliated Hospital of Sun Yat‑Sen University, Shenzhen, Guangdong 518107, P.R. China.; 2 Department of Gastrointestinopancreatic Surgery, The First Affiliated Hospital of Sun Yat‑Sen University, Guangzhou, Guangdong 510080, P.R. China.; 3 Department of Intervention, The People's Hospital of Guangxi Zhuang Autonomous Region,Nanning Guangxi 530021,P.R. China.; 4 Adult Stem Cell Laboratory, The Francis Crick Institute, 1 Midland Road, London NW1 1AT, UK.; 5 The Institute of Cancer Research, 237 Fulham Road, London SW3 6JB, UK.; 6 Animal Experiment Center, The First Affiliated Hospital of Sun Yat-Sen University, Guangzhou 510080, P.R. China.; 7 Scientific research center, The Seventh Affiliated Hospital of Sun Yat‑Sen University, Shenzhen, Guangdong 518107, P.R.China.

**Keywords:** Ailanthone, Gastric cancer, P23, Organoids, BER

## Abstract

Chemotherapy plays an irreplaceable role in the treatment of GC, but currently available chemotherapeutic drugs are not ideal. The application of medicinal plants is an important direction for new drug discovery. Through drug screening of GC organoids, we determined that ailanthone has an anticancer effect on GC cells *in vitro* and *in vivo*. We also found that AIL can induce DNA damage and apoptosis in GC cells. Further transcriptome sequencing of PDX tissue indicated that AIL inhibited the expression of XRCC1, which plays an important role in DNA damage repair, and the results were also confirmed by western blotting. In addition, we found that AIL inhibited the expression of P23 and that inhibition of P23 decreased the expression of XRCC1, indicating that AIL can regulate XRCC1 via P23. The results of coimmunoprecipitation showed that AIL can inhibit the binding of P23 and XRCC1 to HSP90. These findings indicate that AIL can induce DNA damage and apoptosis in GC cells. Meanwhile, AIL can decrease XRCC1 activity by downregulating P23 expression to inhibit DNA damage repair. The present study sheds light on the potential application of new drugs isolated from natural medicinal plants for GC therapy.

## Introduction

Gastric cancer (GC) is the 5th most common cancer worldwide, with a 5-year survival rate of 29%, and it is the 3^rd^ leading cause of cancer-related death. Currently, the most effective treatment is known to be surgical intervention. However, because most GC patients are diagnosed in the late stage of the disease, the window of opportunity for surgery has often passed. For advanced cancer, chemotherapy and perioperative chemotherapy or adjuvant chemoradiotherapy are critical for improving patient survival. Nevertheless, chemotherapy drugs have toxic side effects, and currently, studies have shown that not all patients are sensitive to traditional chemotherapeutic drugs 3. Thus, developing a new, effective, yet low-toxicity drug is of utmost importance for these patients.

Medicinal plants have a long history of application as cancer treatments 4, 5. Currently, many drugs used in clinical settings are derived from medicinal plants. For example, vincristine, paclitaxel and docetaxel are commonplace plant-derived drugs in modern clinical practice 6, 7. Therefore, the identification of natural compounds from medicinal plants and the development of new anticancer drugs from these compounds has been gaining increasing traction over recent years. Here, we combined literature review, organoid drug screening and PDX model verification to identify potential new, effective drugs. Fortunately, AIL turned out to be an effective compound for suppressing GC organoid growth. AIL is a medicinal plant extracted from Ailanthus altissima. It has been used in traditional Chinese medicine (TCM) to treat several diseases since ancient times. In recent years, AIL has been confirmed by modern scientific methods to have significant anticancer activity 8-18. AIL can inhibit cell proliferation and induce apoptosis by up-or downregulating the expression of cancer-associated molecules, which ultimately leads to cancer cell death. In the GC PDX model, AIL was verified to be a better suppressor of tumor growth with lower cell toxicity. Transcriptome sequencing of the PDX tissue indicated that AIL inhibited the expression of XRCC1, which plays an important role in DNA damage repair. Further analysis revealed that AIL can effectively induce DNA damage in GC cells and downregulate P23 expression, which suppresses DNA damage repair in the base excision repair (BER) pathway by regulating XRCC1 activity. P23 is known as an important co-chaperone of HSP90 19.

In this study, we found that AIL can significantly inhibit tumor growth in GC cells, organoids and PDX models both *in vivo* and *in vitro*. AIL mainly inhibited the DNA repair activity of XRCC1 by inducing DNA damage and downregulating P23 expression, thereby exerting an anticancer effect in GC. This finding indicated that AIL could effectively kill tumors through promoting DNA damage and inhibiting DNA repair.

## Methods and Materials

### Extraction of AIL

AIL is extracted from Ailanthus altissima. The extraction method is as follows: As raw material, AIL is initially crushed, and then extracted by mixing 6-12 times the amount of 75-90% ethanol via sono-extraction. The extract was then kept warm and decolorized with an appropriate amount of diatomite until condensed. Using polyamide resin column, the solution was enriched and later eluted with 80% ethanol solution. The eluent was subsequently left to be concentrated and dried. Finally, the dried material was processed using high-speed countercurrent chromatography, resulting in AIL as the final product.

AIL is synthesized in Chengdu HerbPurify Co., Ltd, and its structure was identified by mass spectrometry (Fig. S1).

### Cell Culture

Human gastric cancer cell lines SGC7901, SNU719, AGS were provided by Nanjing Kegen Biotechnology Co., Ltd. The cells were cultured in RPMI-1640 medium containing 10% fetal bovine serum and 1% penicillin and streptomycin, placed in a humidified incubator containing 5% CO^2^ and 95% air, subculture and all experiments were performed at 37°C. AIL stock solution was prepared with DMSO, stored at 4°C, and diluted with RPMI-1640 medium to the required concentration immediately before use; the final concentration of DMSO in the medium was 0.1%. Cells in the control group were treated with DMSO (0.1%) without AIL.

### Human tissues and organoids

Human gastric cancer tissues were taken from patients who underwent gastric cancer surgery in the First Affiliated Hospital of Sun Yat-sen University. They agreed and signed a donation and research consent form. It was approved by the clinical scientific research and animal experiment ethics committee of the First Affiliated Hospital of Sun Yat-sen University (Lunshen [2018] No. 087). This research complied with all the human participation ethics in research. Organoid related experiments were completed in the Digestive Diseases Center of the Seventh Affiliated Hospital of Sun Yat-sen University. Organoid strains from a total of 3 patients were used.

After the tumor was excised, it was placed in 50 U/ml penicillin-streptomycin (Thermo Fisher) frozen G solution. The tissue was minced on ice and incubated in DMEM containing 1 mg/ml collagenase V (Sigma-Aldrich) for 1 hour at 37 °C. Iced PBS was added to stop the digestion, and subsequently centrifuged at 4 °C (300 rcf, 5 min). The sample was further digested with TrypLE (Thermo Fisher) at 37 °C for 5 min, and then stopped with a large amount of PBS. The suspension was filtered through 40 nylon meshes, centrifuged, and the cells were fixed in the matrix. It was then passaged with TrypLE every 2 weeks. The medium for establishing and culturing human GC organoids was as described in the literature 20.

### Colony Formation Assay and Cell Viability

AGS, SNU719, and SGC7901 cells were seeded in 6-well plates with 500 cells per well, and the experiment was repeated three times. AIL was added to the cells at different final concentrations (0, 0.05, 0.1 μM) and it was then incubated for 12 days. The cells were fixed for 30 minutes with 4% paraformaldehyde, stained with crystal violet for 30 minutes, and finally counted.

In a 96-well transparent bottom black board, 3,000 cells were seeded into each well (organoids were seeded in Matrigel). Medications were added in each well according to a 5-fold or 10-fold concentration gradient. After 72 hours, a luminometer (PerkinElmer Life and Analytical Sciences, Boston, MA) was used to determine the level of adenosine triphosphate (ATP) by CellTiter-Glo Luminescent Cell Viability Assay (Promega, Madison, WI).

### Spheroid Colony Formation Assay

The specific method of the spherical colony formation test was as described above 21. Human GC cells are sown in wells (500 cells/or indicated) in ultra-low-attachment 24-well plates supplemented with 2 ml DMEM/F12 medium (glico) and 10 mM HEPES, recombinant human epidermal growth factor (EGF) (Invitrogen) at a concentration of 20 ng/ml, and human recombinant basic fibroblast growth factor (bFGF) (Invitrogen) at a concentration of 10 ng/ml. After 2 to 4 weeks, each well was inspected with a light microscope, and the spherical colonies were counted in 5 random fields of view.

### Cell migration and invasion assay

Transwell migration assay and matrix invasion assay were used to determine cell migration and invasion ability. In the Transwell migration assay, a small chamber (8 µm pore size; Corning) was placed in a 24-well plate, and then 5×10^4^ cells were suspended in 200ul serum-free RPMI-1640 medium in the chamber; 20% pre-warmed fetal bovine serum was used in the well.The cells were incubated for a period of time (AGS/16 hours, SNU719/24 hours, SGC7901/42 hours) in 5% CO^2^ at 37 °C, and then fixed with 4% paraformaldehyde. In the matrix invasion assay, matrix gel and serum-free 1640 medium were injected into the transwell chamber at a ratio of 1:8 for pretreatment for 2 hours. 1×10^5^ cells was suspended in 200 μL of serum-free RPMI-1640 medium, and pre-warmed medium containing 20% fetal bovine serum was then added to the well. The cells were incubated for a period of time (AGS/16 hours, SNU719/24 hours, SGC7901/42 hours) in 5% CO^2^ at 37 °C, and then fixed with 4% paraformaldehyde. A cotton swab was used to gently remove cells that have not migrated or invaded the membrane. Cells were stained with 0.1% crystal violet (Sigma, St Louis, MO), and cells in 8 randomly selected field of vision were counted under a light microscope (100x magnification) to determine cell migration or invasion.

### The PDX mouse model

*In vivo* experiments had been performed according to the Institutional Animal Care and Use Committee (IACUC). It was approved by the clinical scientific research and animal experiment ethics committee of the First Affiliated Hospital of Sun Yat-sen University (Lunshen [2018] No. 087). The experiment was conducted in the Animal Center of the First Affiliated Hospital of Sun Yat-sen University.

Balb/c nude mice (female, 8 weeks old, 19-21g in mass) were purchased from GemPharmatech Co., Ltd (Nanjing, Jiangsu) Experimental Animal Co., Ltd. and raised in an SPF environment. PDX method was as mentioned before 22. When the tumor volume reached about 150 mm^3^, they were randomly divided into three groups, and different reagents were given by intraperitoneal injection (IP). The tumor volume and body mass were measured every 3 days. The calculation formula of tumor volume is as follows: V=Πa b2/8 (where V is the tumor volume, 'a' is the largest tumor diameter, and 'b' is the smallest tumor diameter). Nude mice were sacrificed on day 30, and tumors were taken for measurement and weighing.

### Biochemistry, histology and immunohistochemical staining

In order to evaluate the toxicity of Ailanthone, mice with subcutaneously transplanted tumor were sacrificed at the end of the experiment, and blood serum, liver and kidney were harvested. Serum alanine aminotransferase (ALT) and aspartate aminotransferase (AST) were measured. Tumor or mouse tissue samples were immediately fixed in 10% neutral buffered formalin for 24 hours, gradually dehydrated in a solution with increasing ethanol content (75, 85, 95 and 100%, v/v), and finally embedded in paraffin blocks. Liver and kidney were fixed by paraffin-embedded formalin and cut into 3 mm sections. H&E staining was performed by the experimental histopathology laboratory in accordance with standard procedures. Observations were made under light microscope (100X magnification). Immunohistochemical (IHC) staining: Collect tissues after fixation, dehydration, embedding and sectioning. After heat-mediated antigen repair in 10 mM sodium citrate buffer (pH 6.2) or Tri-EDTA, 1.6% hydrogen peroxide was used to block endogenous peroxidase. Ki67(1: 1000, Abcam, ab15580), H2AX (1: 100, Proteintech, 10856-1-AP), Caspase3 (1:200, Proteintech, 19677-1-AP), XRCC1 (1: 100, Abcam, ab44830), P23 (1: 200, Proteintech, 10824-1-AP), HSP90 (1: 400, Proteintech, 60318-1-Ig, Mouse), and AKT (1: 500, Proteintech, 10176-2-AP) were used as a primary antibody for immunohistochemistry tests according to the protocol. The samples were stained with hematoxylin (HE) and eosin to indicate the nucleus and cytoplasm, respectively. DAB staining was performed and positive cells were counted in 5 random field of views per slide.

### Quantitative real-time PCR

AGS, SNU719, and SGC7901 cells were sown in 6-well plates and incubated with different concentrations of AIL for 12 hours. AG RNAex Pro reagent (Accurate Biology, CAT#AG21102) was used to extract total RNA from tissue samples or cell lines. Evo M-MLV reverse transcriptase premix (Accurate Biology CAT#AG11706) was used to synthesize cDNA from 2 μg RNA of each sample. SYBR Green Premix Pro Taq HS qPCR kit (Accurate Biology, CAT# AG11701) was used for qRT-PCR. Each sample was tested 3 times. The data was analyzed using the 2-ΔΔCT calculation method. The primers were as follows:

- GAPDH forward 5'-GACCCCTTCATTGACCTCAA-3';
- GAPDH reverse 5'-TGCTTCACCACCTTCTTGAT-3';
- HSP90 forward: 5'-CATAACGATGATGAGCAGTACGC-3';
- HSP90 reverse: 5'- GACCCATAGGTTCACCTGTGT-3';
- P23forward: 5'- GAAAGCACAGTAATCACTGGTGT-3';
- P23 reverse:5'- ACGGTAGTCCAATAGAGCAACC-3';
- XRCC1 forward: 5'- TCAAGGCAGACACTTACCGAA-3';
- XRCC1 reverse: 5'- TCCAACTGTAGGACCACAGAG-3'.

### Western blotting

The cells were processed differently according to their corresponding requirements, and the protein concentration is determined by BCA Protein Assay Kit (KGI Biosciences), using SDS-PAGE Sample Loading Buffer, 5X (Beyotime) at 95° for 8 minutes. The lysate is separated on a polyacrylamide gel and transferred to nitrocellulose. The blot was detected with a specific antibody, and then the membrane was detected with the LI-COR Odyssey CLx Infrared Imaging System (LI-COR Biotechnology, Lincoln NE). Antibodies were γH2AX (1: 1000, Proteintech, 10856-1-AP), XRCC1 (1: 1000, Abcam, ab44830), Caspase3 (1: 400, Proteintech, 19677-1-AP), P23 (1: 500, Proteintech, 10824-1-AP), AKT (1: 2000, Proteintech, 10176-2-AP), HSP90 (1: 1000, Proteintech, 60318-1-Ig), Tubulin (1: 1000, Proteintech, 10068-1- AP), GAPDH (1: 20000, Proteintech, 60004-1-Ig).

### Immunoprecipitation

The protein expression level was determined by the western blotting results of cytoplasm or nuclear lysate. Nuclear and Cytoplasmic Protein Extraction Kit (Beyotime, CAT#P0028) was used to prepare cytoplasmic lysates and nuclear lysates, and protein concentration was measured. 2,000 μg of protein lysate (prepared 500 μL) was mixed with primary antibody and incubated overnight in a 4° shaker. 50 μL of protein A/G agarose (Thermo Fisher Scientific, CAT#20424) beads was washed 3 times with lysate, mixed in the above-mentioned protein mixture with the primary antibody and incubated overnight in a 4° shaker. The mixture was washed 6 times with lysis buffer (Thermo Fisher Scientific, CAT#87787) before SDS-PAGE Sample Loading Buffer was added, and then heated to 95° for 10 min at 5X (Beyotime), centrifuged at 3,000 rpm for 3 min, the supernatant was carefully aspirated and the protein level was finally detected via western blotting. Antibodies used were: XRCC1 (1: 100, Abcam, ab44830), P23 (1: 200, Proteintech, 10824-1-AP), HSP90 (1: 400, Proteintech, 60318-1-Ig), Lamin B1 (1: 20000, Proteintech, 66095-1-Ig), GAPDH (1: 20000, Proteintech, 60004-1-Ig).

### Immunofluorescent (IF) staining

The collected organoids were fixed for 16-20 hours, then embedded, sliced, baked, and deparaffinized. After heat-mediated antigen retrieve in 10 mM sodium citrate buffer (pH 6.2) or Tri-EDTA, 1.6% hydrogen peroxide was used to quench endogenous peroxidase, then blocked with PBS containing 5% BSA and 0.2% Triton- X. The primary antibody was incubated in blocking buffer at 4°C for 16-20 hours. The fluorescent secondary antibody was incubated for 1 hour at 20°C, and then incubated in DAPI for 10 minutes. Fluorescence staining was imaged on a Zeiss LSM 780 confocal microscope. Primary antibodies used were: Ki67 (1: 1000, Abcam, ab15580), H2AX (1: 400, Proteintech, 10856-1-AP), Caspase3 (1: 400, Proteintech, 19677-1-AP ), XRCC1 (1: 100, Abcam, ab44830), P23 (1: 200, Proteintech, 10824-1-AP), HSP90 (1: 400, Proteintech, 60318-1-Ig), AKT (1: 400, Proteintech, 10176-2-AP). Secondary antibodies used were: Donkey anti-Rabbit/Alexa Fluor 488 (1:1000, Thermo Fisher Scientific, A-21206), Donkey anti-Rabbit/Alexa Fluor 546 (1:500, Thermo Fisher Scientific, A11040), Donkey anti-Mouse/Alexa Fluor 488 (1:000, Thermo Fisher Scientific, A-21202), Donkey anti-Mouse/Alexa Fluor 546 (1:500, Thermo Fisher Scientific, A10036).

### RNA isolation and RNA sequencing

Total RNA was extracted from tissue samples, the concentration and purity of the extracted RNA were detected by Nanodrop 2000, RNA integrity was detected by agarose gel electrophoresis, and RIN value was determined by Agilent 2100. A single library construction requires that the total amount of RNA is not less than 5 μg, concentration ≥ 200 ng/μL, and the OD260/280 is between 1.8 and 2.2. mRNA capture and library preparation were completed by the advanced sequencing equipment of Shanghai Origingene Bio-pharm Technology Co., Ltd. using KAPA mRNA HyperPrep kit (Roche). Three biological library was sequenced on the Illumina Truseq TM RNA sample prep Kit platform of the facility, and each sample produced an average of 25 million single-ended reads of 75 bp. The high-quality DNA sequencing obtained after quality control was compared with the designated reference genome. For the PDX sample, it was first compared to the mouse reference genome. After eliminating the mouse data, the remainder was then compared to the human reference genome retrieved from Ensembl database (genome version GRCh38, gene annotation information Ensemble 92). Before alignment, cutadapt (version 1.9.1) was used for quality trimming and adaptor removal of the original reading. Using the annotation release 86 as a reference, read was sequenced on human genome GRCh38 using RSEM 1.3.0 and STAR 2.5.2, and the subsequent gene levels were counted. In R program (version 3.6.1), the DESeq2 package (version 1.24.0) was used for normalization and differential expression analysis of raw count data. Regularized logarithmic transformation was performed on the rlog function.

### Gene Set Enrichment Analysis (GSEA)

GSEA was performed with the software (GSEA V4.0.3) developed by the Broad Institute of Massachusetts Institute of Technology and Harvard University (https://www.gsea msigdb.org/gsea/index.jsp). For the RNA-seq datasets of low and high expressionof P23, OXA group and AIL group, the normalized RNA read count was used for analysis, and the following settings were applied: number of permutations = 1000, type of permutation = gene set, enrichment statistics = weighting, measurement of gene ranking = signal noise. For the TCGA gastric cancer data set, the samples were grouped according to their expression above or below the median value. The normalized RSEM read count was used for analysis, and the following settings were applied: number of permutations = 1000, permutation type = phenotype, enrichment statistics = weighting, measurement of gene ranking = signal 2 noise. Recognized marker gene set 40, KEGG pathway or gene ontology (GO) terms and false discovery rate (FDR q) <0.05 were set as significant enrichment.

### Screening of differentially expressed genes (DEGs)

The expectation-maximization algorithm of RNA-Seq was used to normalize the 3-level transcriptome data of the data set, and the logarithmic transformation of all gene expression values was performed. Approximate data were normalized by quantiles and were normally distributed 23. In this study, the R program package limma v3.28.14 was used to analyze the differential genes in the gene expression data, and its mRNA satisfied P<0.01, false discovery rate (FDR) was <0.01 and |log2 fold change (FC)| was >1.5, where P <0.05 indicated that the hypothesis test was statistically significant. FDR is a control indicator for the error rate of the hypothesis test. As an evaluation index of the selected differential genes, the number of false rejections was proportional to the number of null hypotheses rejected. FC is usually used to describe the degree of change from the initial value to the final value. In this study, the ratio of tumor tissue gene expression value to normal tissue gene expression value was used, also known as the fold change. The heatmap and volcano map of the differential genes were constructed in R language for visual comparison.

### Flow cytometry and FACS

Flow cytometric analysis: Single cells prepared from organoids was suspended in PBS containing 10% FBS. The single cell suspension was stained with a fluorescently-labeled isotype control antibody or experimental antibody in the dark at 4 °C for 30 minutes, and washed three times in FACS buffer. The cells were filtered through a 40-HCLM nylon mesh and incubated in 3 HCLM DAPI in FACS buffer. Single-color stains and Fluorescence Minus One control were used when necessary. Annexin V-PI apoptosis assay and cell-cycle analysis were performed using Annexin V-FITC apoptosis detection kit (KeyGEN BioTECH, KGA105) and apoptosis detection kit (KeyGEN BioTECH, KGA512) according to the manufacturer's protocol. Cells were sorted and analyzed by CytoFlex LX (Beckman). FlowJo 10 software was used to analyze the data.Flow sorting: After the prepared organoid suspension single cell was fixed (Cytofix/Cytoperm Soln Kit, CAS: 554714), P23 was added (1:100, Thermo Fisher Scientific, MA3-414) and incubated at 4° for 30 min, then washed three times in FACS buffer, incubated with the fluorescent secondary antibody Donkey anti-Rabbit/Alexa Fluor 488 (1:200, Thermo Fisher Scientific, A-21206) for 15 minutes, and lastly dead cells were excluded after staining with DAPI. The cells were sorted on a SH800S (SONY) flow cytometer, and P23(+) and P23(-) cell populations of the GC organoid strains were separated by flow cytometry. The cells were collected in cold PBS supplemented with 10% fetal bovine serum, and the final sorted cell population was subjected to other experiments.

### Clinical gastric cancer samples

The sample data were procured from the prospectively collected GC database of the First Affiliated Hospital of Sun Yat-sen University, Guangzhou, China. We reviewed the treatment records of patients undergoing gastric cancer resection from January 2010 to May 2012. The inclusion criteria were pathological TNM stages I-III gastric adenocarcinoma patients whom underwent radical surgery patients. Exclusion criteria: other history of malignant tumors, neoadjuvant therapy, loss to follow-up, no tissue samples. A total of 93 cases of tissue samples were collected from surgical specimens, and informed consent was obtained from the patients. This study was approved by the Ethics Committee of the First Affiliated Hospital of Sun Yat-sen University. The detailed clinical characteristics of the patients were shown in Table 1. The endpoint of the study was recurrence. The specimen tissues were tested by IHC, and the results were interpreted by two independent pathologists. They were blinded to the specific diagnosis and prognosis of each case, and used a semi-quantitative method for scoring. Among the samples, more than 10% of tumor cells staining were positive. The staining intensity was divided into negative, weak, moderate and strong. Low P23 expression was detected in the negative and low intensity samples, and medium and strong intensity returned with high P23 expression. Subsequently, the disease-free survival of patients in the high P23 expression group (n=42) and low P23 expression group (n=51) were evaluated. Progression free survival is defined as the duration from tumor resection to progressive disease. Follow up was completed in intervals of 3 months (0-2 years), 6 months (2-4 years), and once a year until recurrence or June 2020. The follow-up study included abdominal computed tomography and postoperative physical examination.

### Statistical analysis

SPSS 22.0 software was used for statistical analysis. Data were expressed as mean±standard deviation (s.d.), and P<0.05 was considered statistically significant. Tumor volume was measured repeatedly using a general linear model. The difference between categorical variables was tested using χ^2^ test, and the difference between two groups was tested using student's t test. Kaplan-Meier curve and log-rank test were used to analyze the clinical data of gastric cancer patients.

## Results

### AIL has a good inhibitory effect on GC

**Figure 1:**
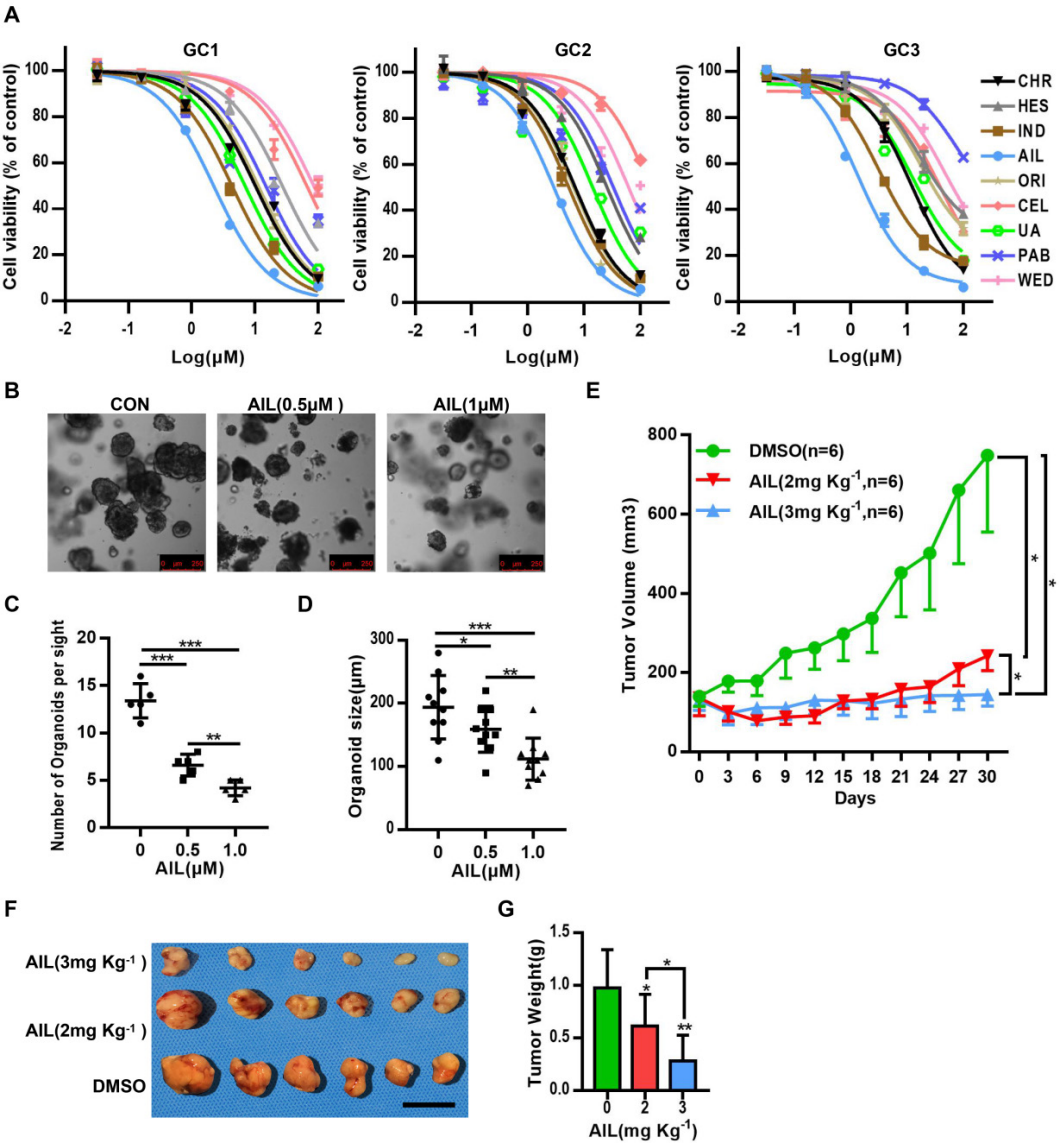
** AIL inhibits the proliferation of GC cells *in vitro* and *in vivo*.** (A) The 9 types of medicinal plants used for drug screening in three GC organoids, GC1, GC2, and GC3. After 72 hours, cell viability was analyzed via CCK8. Data were derived from experiments conducted in triplicate. (B-D) Representative images of GC organoids treated with DMSO (control group) and specified concentrations of AIL for 14 days. The number of cell clusters (C) and size of clusters (D) were counted. Data were derived from experiments conducted in triplicate. (E-G) PDX models were established with subcutaneous inoculation of nude mice with human-derived GC tissue. After the tumors grew to a certain volume, the mice were randomly divided into 3 groups (6 mice in each group) and received daily intraperitoneal injections of DMSO or AIL (2 mg/kg or 3 mg/kg) for 30 days. (E) Tumor volume was measured every three days. (F) Subcutaneous tumor tissue from 3 experimental groups of nude mice. (G) Tumor weight statistics of subcutaneous tumor tissue from 3 experimental groups of nude mice. The data are expressed as the mean±SD of 6 mice (n=6) in each group. Scale bars, 10 mm. These mice were compared with the control group, *P<0.05, **P<0.01, ***P<0.001. AIL: ailanthone; CEL: celastrol; ORI: oridonin; HES: hesperidin; CHR: chrysin; IND: indirubin; UA: ursolic acid; WED: wedelolactone; PAB: pseudolaric acid B.

Many drugs developed to date are monomers that were first inspired by, and subsequently extracted from, plants, such as vincristine, which has been widely used for the clinical treatment of cancer 7, and artemisinin, which saves countless lives from malaria 7. From the above, it is clear that seeking inspiration from ancient methodologies such as TCM is an important approach for discovering new medicines. From the 166 types of medicinal plant with anticancer effects recorded in the Pharmacopoeia of the People's Republic of China (2000 edition) 25, we selected 9 types of medicinal plant (Table 1) that have been suggested through modern science approaches in recent years to have relatively good anticancer effects (Figure S2A). We then conducted drug susceptibility tests on three strains of GC organoids (Figure S2B-D). Fortunately, AIL excelled above the tested compounds (Figure 1A). AIL is an herbal monomer extracted from the bark of Ailanthus altissima 26, which in recent years has been proven to have obvious anticancer effects on multiple cancer types. To verify whether AIL has the potential to inhibit the growth of GC cells, we evaluated its effect on GC cell growth and colony formation. The results showed that AIL significantly inhibited the growth of the AGS, SNU719 and SGC 7901 cell lines (Figure S3A,B). We also evaluated its effect on the spheroidizing ability of GC organoids, and the results showed that AIL significantly inhibited the spheroidizing ability of GC organoids (Figure 1B-D). We further verified the effect of AIL on GC PDX models and found that it also had a good inhibitory effect *in vivo* (Figure 1E-G, Figure S4A,B). There was no significant weight loss observed in the mice during treatment (Figure S5A) and no significant difference in serum ALT and AST levels posttreatment (Figure S5B). No negative impact was observed on liver or kidney function (Figure S5C). Additionally, AIL exerted a significant inhibitory effect on GC cell migration and invasion in transwell cell migration and invasion assays (Figure S6A-D). In summary, these results indicated that AIL could significantly inhibit GC cells *in vitro* and *in vivo* while maintaining low cell toxicity. However, the mechanism by which AIL inhibits the proliferation of GC cells is unknown; thus, we further explored its mechanisms.

### AIL promotes apoptosis by inducing DNA damage

**Figure 2:**
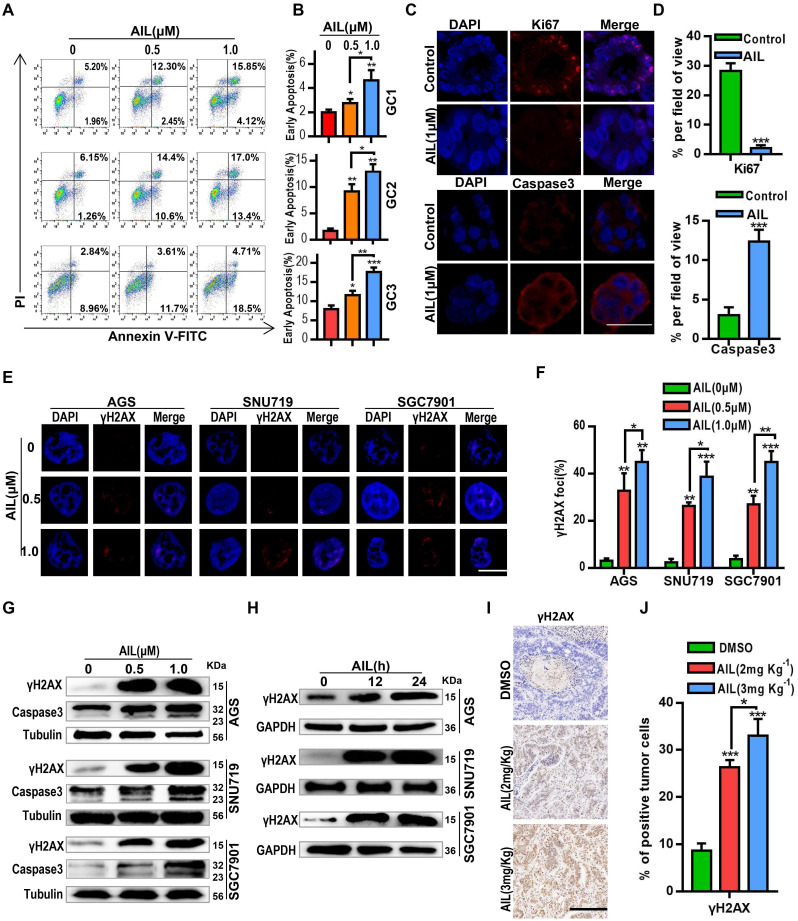
** AIL induces DNA damage and apoptosis in GC cells.** (A) Three GC organoids were treated with DMSO (control group) and specified concentrations of AIL for 48 hours, followed by digestion into single cells and analysis of apoptosis through flow cytometry analysis with FITC-Annexin V and PI staining. (B) Data were derived from experiments conducted in triplicate in (A). (C) GC organoids were embedded and sectioned after treatment with DMSO (control group) and AIL (1 µM) for 48 hours. An immunofluorescence assay was performed to analyze the expression of Ki67 and Caspase3. Scale bars, 100 µm. (D) Data were derived from experiments conducted in triplicate in (C). (E) Representative images of γH2AX foci in AGS, SNU719 and SGC7901 cells treated with specified concentrations of AIL for 24 hours. Scale bars, 5 µm. (F) Data were derived from experiments conducted in triplicate in (E). (G) Western blotting was performed to detect the expression levels of γH2AX and Caspase3 in GC cell lines (AGS, SNU719 and SGC7901) treated with specified concentrations of AIL for 24 hours. (H) AGS, SNU719 and SGC7901 cells were treated with AIL (0.5 µM) for 0, 12, and 24 hours, and the expression level of γH2AX was detected by western blotting. (I) Representative images of γH2AX levels in PDX tumor tissues from nude mice through immunohistochemistry (100X). Scale bars, 200 µm. (J) Data were derived from experiments conducted in triplicate in (I). They were compared with the control group, *P<0.05, **P<0.01, ***P<0.001.

To determine how AIL inhibits the proliferation of GC cells, we conducted a series of exploratory experiments. Apoptosis analysis was performed via flow cytometry on GC organoids after AIL treatment. The results showed that AIL significantly induced apoptosis (Figure 2A,B). We also explored whether AIL increases DNA damage in GC cells. Western blotting and IF analysis found that AIL induced DNA damage in GC cells (Figure 2C-H) in a concentration- (Figure 2E-G) and time-dependent manner (Figure 2H). AIL had a stronger ability to induce DNA damage (Figure 2C-J). We supplemented data on DNA damage and apoptosis of immunofluorescence on organoids by AIL on the 0, 12 and 24 h mark (Figure S7A,B), and found that AIL induced DNA damage in gastric cancer at 12, 24 h was similar, while AIL induced apoptosis did not significantly increase at 12 h, but slightly increased at 24 h. This suggests that AIL-induced apoptosis occurs after DNA damage and is time-dependent. Thus, we speculate that AIL may be able to inhibit DNA repair, thereby causing continuous DNA damage.

### AIL inhibits DNA repair in GC cells

**Figure 3:**
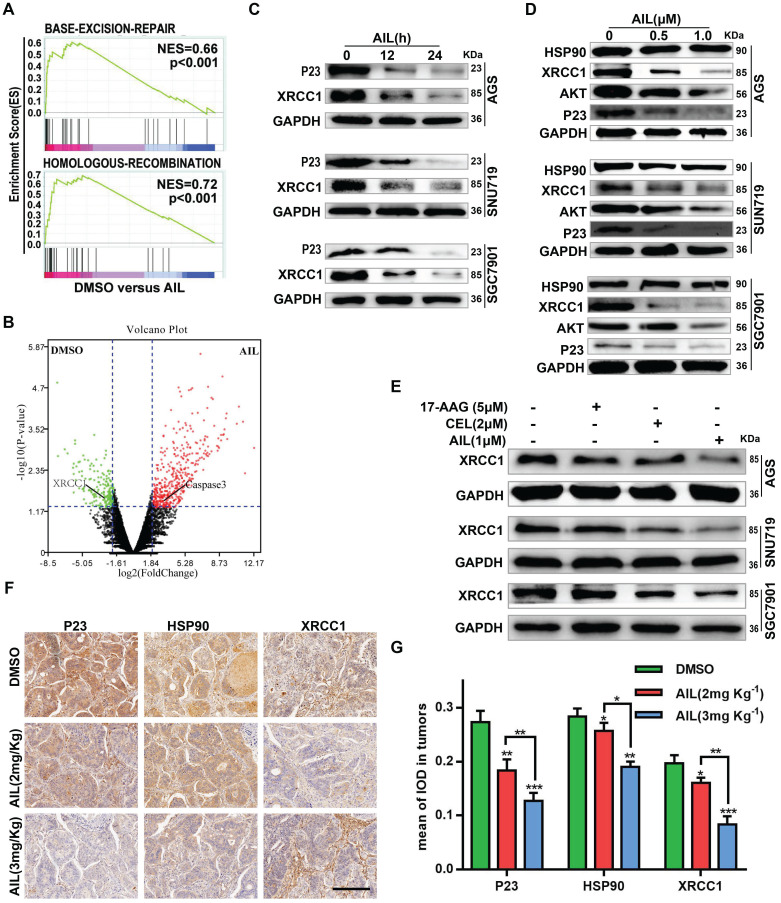
** AIL inhibits DNA damage repair of GC cells.** (A) Transcriptome sequencing and pathway enrichment were performed on PDX tumor tissues from DMSO- and AIL-treated mice. (B) Differential gene expression analysis of the transcriptome sequencing. (C) AGS, SNU719 and SGC7901 cells were treated with AIL (0.5 µM) for 0, 12, and 24 hours, the cells were then lysed, and the expression levels of P23 and XRCC1 were detected by western blotting. (D) Western blotting was performed to analyze the expression of HSP90, XRCC1, AKT and P23 in GC cell lines (AGS, SNU719 and SGC7901) after treatment with the specified concentrations of AIL for 24 hours. (E) AGS, SNU719 and SGC7901 cells were treated with 17-AAG (HSP90 inhibitor, 5 µM), CEL (P23 inhibitor, 2 µM) and AIL (1 µM) for 24 hours, and the expression of XRCC1 was detected by western blotting. (F) Representative images of P23, HSP90, and XRCC1 in PDX tumor tissue as detected via immunohistochemistry (100X). Scale bars, 200 µm. (G) Data were derived from experiments conducted in triplicate in (F). The PDX tumor tissues were compared with the control group, *P<0.05, **P<0.01, ***P<0.001.

To test the above hypothesis, we sequenced the transcriptomes of PDX model tissues. The pathway enrichment analysis revealed that AIL primarily regulated the BER DNA repair pathway (Figure 3A); the differential gene expression analysis suggested that AIL had a significant inhibitory effect on the key BER pathway gene XRCC1 (Figure 3B) 27-29. Subsequent experiments confirmed that AIL has a good inhibitory effect on XRCC1 in GC cells (Figure 3-C,D,F,G). In a previous study, AIL was proven to treat castration-resistant prostate cancer by downregulating P23 expression 17, 30. Bioinformatics analysis showed that P23 is closely related to DNA repair-related pathways; that is, high expression of P23 leads to increased enrichment of the BER, HR, NHEJ, and MMR pathway genes than low expression of P23, indicating that P23 promotes DNA repair (Figure S8A). Western blotting also confirmed that AIL can significantly inhibit the expression level of P23 in GC cells (Figure 3C,D). The IF analysis of organoids also confirmed that AIL inhibits the expression of P23 and XRCC1 (Figure S9A). As an important co-chaperone of HSP90, P23 plays a key role in HSP90's participation in chromatin remodeling, DNA transcription, RNA processing, DNA replication, telomere maintenance and DNA repair. In fact, the involvement of HSP90 in DNA repair requires its co-chaperone P23 or phosphorylation to interact with DNA metabolism proteins. However, our results showed that AIL did not increase the expression of HSP90 (Figure 3D). Instead, we noted that AIL is not an ATP-competitive inhibitor of HSP90, unlike the HSP90 inhibitor 17-AAG, which increases the expression of HSP90 protein 31. Interestingly, AIL sufficiently inhibited AKT protein expression in GC (Figure 3D). We further compared the inhibitory effects of 17-AAG, CEL (a P23 inhibitor) and AIL on XRCC1 and found that AIL has significant advantages over the other two compounds (Figure 3E). IHC of PDX tissue also confirmed that AIL inhibited the expression of P23, HSP90, XRCC1 and AKT (Figure 3F,G; Figure S3A,B). These results indicated that the DNA damage of GC cells induced by AIL was mainly related to the inhibition of the BER pathway.

### AIL regulates XRCC1 activity through P23 to inhibit DNA repair

**Figure 4:**
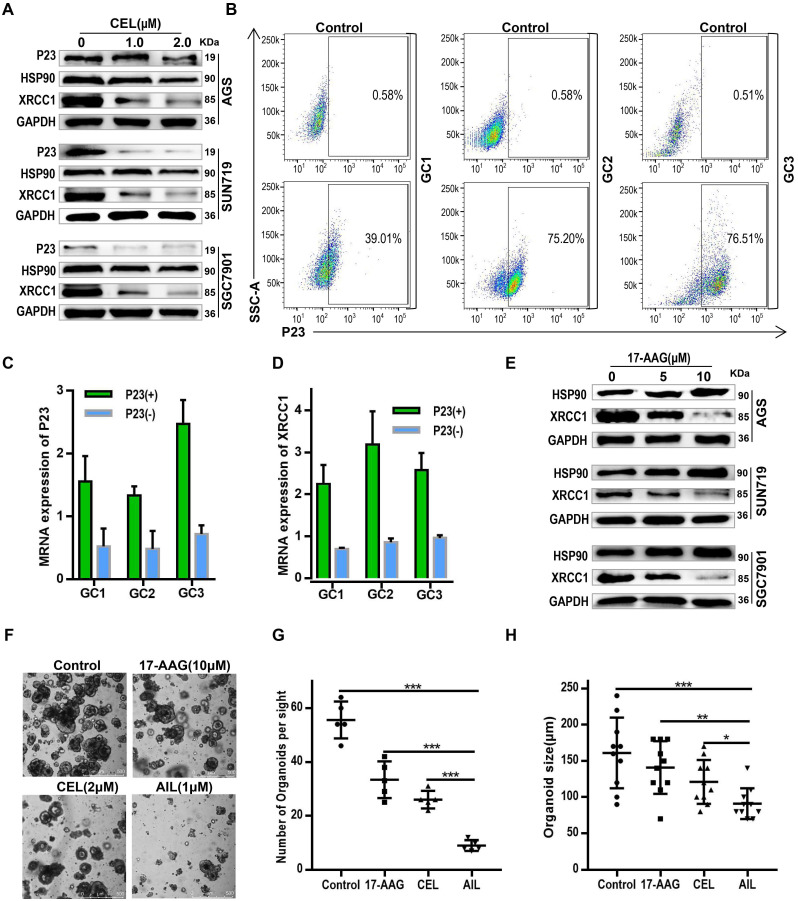
** AIL regulates XRCC1 through P23 to inhibit DNA damage repair.** (A) Western blotting was performed to detect the expression of P23, HSP90 and XRCC1 in AGS, SNU719 and SGC7901 cells after treatment with different concentrations of CEL (P23 inhibitor) for 24 hours. (B) GC1, GC2, and GC3 organoids were flow-sorted to separate P23(-) and P23(+) organoid strains. (C) qPCR was performed to detect P23 mRNA levels in P23(-) and P23(+) organoid strains. (D) qPCR analysis of XRCC1 expression in P23(-) and P23(+) organoid strains. (E) Western blot analysis of the expression of HSP90 and XRCC1 in AGS, SNU719 and SGC7901 cells treated with the specified concentrations of 17-AAG (HSP90 inhibitor) for 24 hours. (F) Representative images of GC organoids treated with DMSO (control group), 17-AAG (10 µM), CEL (2 µM), and AIL (1 µM) for 14 days. The number of cell clusters (G) and size of clusters (H) were counted. Data were derived from experiments conducted in triplicate. The treated organoids were compared with the control group, *P<0.05, **P<0.01, ***P<0.001.

Although we have confirmed the effects of AIL on P23, HSP90, and XRCC1, the regulatory relationship among these proteins is still unclear. HSP90 and its associated chaperone proteins have important functions in the nucleus, including chromatin remodeling, DNA transcription, RNA processing, DNA replication, telomere maintenance and DNA repair 32, 33. The involvement of HSP90 in DNA repair is dependent upon its co-chaperone P23 or phosphorylation, which allow it to interact with DNA metabolism proteins 32-34. As an important HSP90 client protein, the scaffold protein XRCC1 efficiently promotes the repair of DNA single-strand breaks (SSBs) 35, 36. If the SSBs are not properly repaired, they may convert into double-strand breaks during DNA replication, eventually leading to genetic instability and apoptosis 37. Therefore, we theorize that AIL inhibits the interaction between P23 and HSP90 by downregulating P23 expression, thereby affecting the interaction between HSP90 and its client protein XRCC1. To verify the regulatory axis of this molecular mechanism, we first proved the regulatory effect of a P23 inhibitor (CEL) on HSP90 and XRCC1 (Figure 4A). To verify that AIL functions through P23, we separated P23(+) GC organoid and P23(-) GC organoid strains through flow sorting (Figure 4B). qPCR analysis revealed that P23(+) GC organoids had upregulated XRCC1 expression, while P23(-) GC organoids had inhibited XRCC1 expression (Figure 4C,D). Subsequently, we verified the inhibitory effect of HSP90 inhibitors on XRCC1 in GC cells (Figure 4E). Thus, we confirmed that AIL inhibited XRCC1 by downregulating P23 expression. Further bioinformatics analysis revealed that HSP90 is positively correlated with DNA repair levels; that is, as HSP90 expression is upregulated, its DNA repair ability increases (Figure S10A). In summary, AIL has an obvious tumor suppressing effect as it can induce DNA damage while also inhibiting DNA damage repair in GC cells. Thus, we further compared the spheroidization ability of GC organoid cells treated with 17-AAG, CEL and AIL, and the results showed that AIL has an excellent anticancer effect. We supplemented the experiment of P23 inhibitor (CEL) to intervene the apoptosis of organoids (Figure S11A,B), and the experimental results showed that CEL induced the apoptosis of gastric cancer organoids. This indicated that P23 was not only upstream of XRCC1 but also regulated XRCC1.

### AIL inhibits the binding of P23 to HSP90 and HSP90 to XRCC1

**Figure 5:**
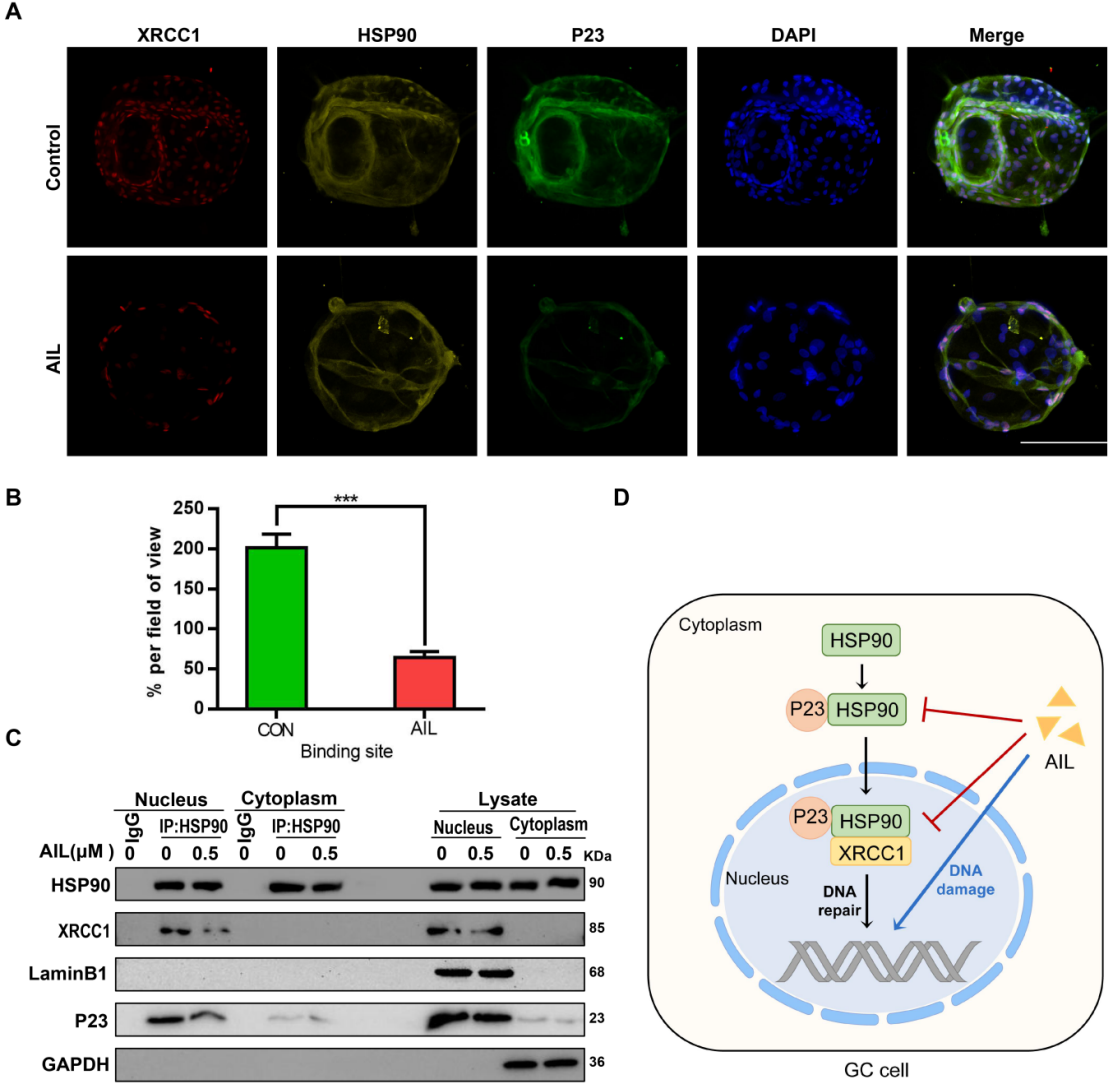
** AIL inhibits the binding of P23 and XRCC1 to HSP90 in GC cells.** (A) The colocalization of HSP90 with P23 and XRCC1 was demonstrated with immunofluorescence in GC organoids treated with AIL (1 µM) for 24 hours. Scale bars, 100 µm. (B) Data were derived from experiments conducted in triplicate in (A). (C) Pull-down of selected proteins with HSP90 in the nucleus and cytoplasm of GC cells treated with AIL for 24 hours, and then the IP fractions were immunoblotted with HSP90, XRCC1, P23, Lamin B1 and GAPDH. (D) Graphical illustration of the functions of AIL in inhibiting tumor growth. The treated cells were compared with the control group, *P<0.05, **P<0.01, ***P<0.001.

As an important co-chaperone of HSP90, P23 binds stably with HSP90 in cancer cells to form a superchaperone complex 38 and can also bind with HSP90 in the absence of any client protein 38; in contrast, XRCC1 requires binding with HSP90 as a chaperone molecule to participate in DNA repair 40. Since AIL can inhibit the expression of P23 and XRCC1, we investigated whether it also inhibits the interaction between P23 or XRCC1 and HSP90. Through immunocolocalization and coimmunoprecipitation, we proved that both P23 and XRCC1 can bind with HSP90 in GC cells and that AIL can inhibit their binding (Figure 5A-C). The above results revealed that AIL not only inhibited the expression of P23 and XRCC1 but also inhibited the binding of P23 to HSP90 and HSP90 to XRCC1.

### High expression of P23 is related to GC recurrence

**Figure 6:**
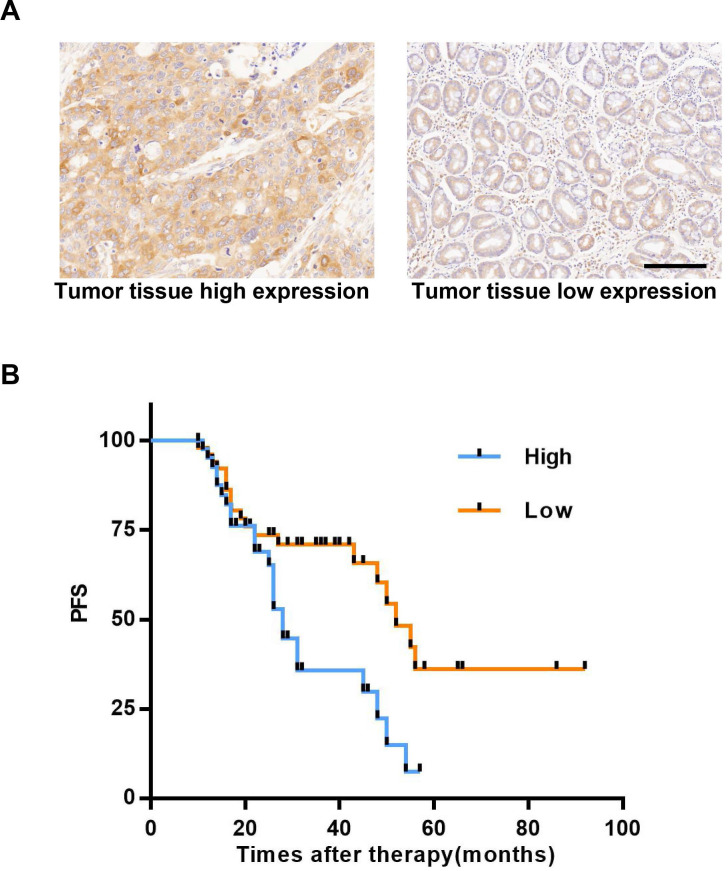
** Highly expressed P23 is associated with GC recurrence.** (A) Representative images of high and low expression levels of P23 detected via IHC (100X) in recurrent GC tissues. Scale bars, 200 µm. (B) Recurrence time curves of 93 patients with GC recurrence, including 42 patients with high P23 expression and 51 patients with low P23 expression. *P<0.05, **P<0.01, ***P<0.001.

P23 has been proven to promote breast cancer metastasis and affect its prognosis 41; P23 also promotes the invasion and metastasis of prostate cancer 41, 42, and targeting P23 can treat castration-resistant prostate cancer 17. In this study, we proved that AIL can target P23 in GC cells. To explore the clinical impact and status of P23 in GC, we enrolled patients with GC recurrence from the Gastric Cancer Research Center of Sun Yat-sen University. We used IHC of tumor tissues to determine the expression level of P23 and found that P23 was highly expressed in the specimens of patients who experienced relapse (Figure 6A). The time to recurrence in patients with high expression of P23 was significantly shorter than that of patients with low expression (Figure 6B). P23 promoted GC recurrence, and P23 could be used as an important clinical indicator to predict patient postoperative disease recurrence.

## Discussion

Chemotherapy plays a pivotal role in GC treatment 2; however, even with a variety of chemotherapeutics and targeted therapies currently available in the clinic, its therapeutic effect is still unsatisfactory. As the fifth most common cancer worldwide, GC is still the third leading cause of cancer-related death, with a 5-year survival rate of only 29% 1. Therefore, it is crucial to find new drugs for GC treatment. In this study, we selected medicinal plants with anticancer effects from the “Pharmacopoeia of the People's Republic of China” (2000 edition), combined with literature meta-screening and application of organoids established by our center as a powerful drug screening tool 43-45, We successfully screened monomeric AIL, which can significantly inhibit the proliferation of GC cells, induce apoptosis, and has a good anticancer effect *in vivo*.

Organoids are multicell clusters constructed by three-dimensional culture *in vitro* that have the ability to self-renew and self-organize, thus maintaining the physiological structure and function of their source tissues 46. With this advantage, organoids quickly became powerful tools for tumor and stem cell biology research. In this study 43, we used organoids as a research tool and confirmed that AIL can effectively inhibit the proliferation of GC cells *in vitro* and *in vivo*. AIL can significantly induce GC cell apoptosis. Through transcriptome sequencing of PDX tissues, we proved that AIL could suppress DNA repair pathways; differential gene expression analysis suggested that AIL had a significant strong inhibitory effect on XRCC1, the key gene in the BER pathway. Subsequent experiments also confirmed that AIL has a good inhibitory effect on XRCC1 in GC cells. Therefore, we speculated that AIL could inhibit DNA damage repair while inducing DNA damage in GC cells.

XRCC1, as an important gene for DNA repair in the BER pathway 27, 47, can interact with PARP1, DNA ligase III and Pol proteins to promote the effective repair of DNA SSBs 35, 36. If SSBs are not repaired promptly and properly, they may convert into DSBs during DNA replication, leading to apoptosis 37. XRCC1 function depends on the binding and stability of HSP90 40. By binding to and stabilizing XRCC1, the phosphorylated form of HSP90 promotes the formation of additional XRCC1 complexes; in the absence of HSP90 or HSP90 binding, free XRCC1 will be removed by ubiquitin-mediated degradation 40. In recent years, increasing evidence has shown that HSP90 plays an important role in cell homeostasis, transcription regulation, chromatin remodeling and DNA repair 32, 33, 47. When HSP90 is involved in DNA damage repair, it depends on P23 to play a role 48. P23 is known to be an important co-chaperone of HSP90 19. P23 not only has overlapping functions with HSP90 but also has other functions independent of HSP90 49. In the past, the HSP90 chaperone system was thought to function in the cytoplasm 50. It is becoming increasingly clear that HSP90 and its associated chaperone proteins have important functions in the nucleus, including chromatin remodeling, DNA transcription, RNA processing, DNA replication, telomere maintenance and DNA repair 29, 30. Our study also revealed that P23 can promote DNA repair. It has been reported that AIL can treat castration-resistant prostate cancer by inhibiting P23 17. Surprisingly, our study also verified that AIL can significantly inhibit P23 in GC cells. AIL can also inhibit the protein binding of P23 to HSP90 and HSP90 to XRCC1. Additionally, AIL can inhibit AKT and the binding of AKT to HSP90. AKT can mediate DNA damage repair 51, and act as a client protein of HSP90 while interacting with HSP90 52. It is possible that the DNA damage induced by AIL itself and its other effect of DNA repair inhibition by blocking multiple signaling pathways of GC cells led to synthetic lethality 52.

P23 plays a key role in DNA repair, and through this study, we confirmed that AIL can significantly inhibit P23 and XRCC1 as effectively as CEL (P23 inhibitors) inhibit XRCC1. Interestingly, dividing GC organoids into P23(+) and P23(-) groups through flow sorting revealed that the P23(+)group had upregulated XRCC1 expression, while the P23(-) group had downregulated XRCC1 expression. All these results indicated that P23 plays a central role in the P23/HSP90/XRCC1 axis. Other studies have revealed that the expression of P23 is increased in some cancers. For example, high P23 expression promotes breast cancer progression and results in poor prognosis by increasing lymph node metastasis and drug resistance 52; while cells with high P23 expression in prostate cancer cells results in a more aggressive phenotype, which promotes invasion and metastasis, thereby leading to disease progression and a worse prognosis 42. However, research on the effect of P23 on the clinical prognosis of GC has not yet been carried out. Our comparative study on the time to recurrence of GC in patients with low to high expression of P23 through immunohistochemical methods revealed that patients with high expression of P23 had a shorter relapse time, indicating that P23 is an important factor affecting the recurrence and development of GC. Therefore, our study provides an important foundation for the targeted therapy of P23.

## Conclusion

In summary, we clarified the anticancer effect of AIL in GC by using GC cell line, organoid and PDX model. It is clear that AIL can not only induce DNA damage but also inhibit DNA repair by downregulating P23 expression, which in turn inhibits the DNA repair capability of XRCC1, thereby exerting a tumor-suppressing effect on GC. This finding indicates that AIL can effectively kill tumors through DNA damage and inhibition of DNA repair. AIL may be used as an effective novel anticancer treatment.

## Supplementary Material

Supplementary figures and tables.

## Figures and Tables

**Table 1:** Research status of 9 medicinal plants

Medicinal plants	Tumor type	References
Chrysin	Prostate cancer, ovarian cancer	365
Hesperidin	Cholangiocyte, oral carcinogenesis	282
Ailanthone	Prostate cancer, lung cancer	25
Indirubin	Prostate cancer, promyelocytic leukemia, glioma	157
Oridonin	Fibrosarcoma, diffuse large B cell lymphoma, leukemia	261
Celastrol	Melanoma, breast cancer, prostate cancer	321
Ursolic acid	Colorectal cancer, breast cancer, leukemia	563
Pseudolaric Acid B	Glioma, lung cancer	59
Wedelolactone	Prostate cancer, pancreatic cancer	45

**Table 2:** Characteristics of 93 recurrence gastric cancer patients

Characteristics		Low	High	*p*
**Sex**				0.419
Female	26	16	10	
Male	67	35	32	
**Differentiation**				0.003
Well	62	41	21	
Moderate	26	7	19	
Poor	5	3	2	
**pT stage**				0.870
T1	22	13	9	
T2a	13	6	7	
T2b	28	14	14	
T3	21	13	8	
T4	9	5	4	
**pN stage**				0.946
N1	58	32	26	
N2	25	14	11	
N3	10	5	5	
**pM stage**				0.851
M0	84	46	38	
M1	9	5	4	

## Abbreviations

AIL: ailanthone
OXA: oxaliplatin
GC: gastric cancer
TCM: traditional Chinese medicine
PDX: patient-derived xenograft
XRCC1: X-ray repair cross-complementing protein 1
BER: base excision repair
BSA: bovine serum albumin
FBS: fetal bovine serum
CEL: celastrol
ORI: oridonin
HES: hesperidin
CHR: chrysin
IND: indirubin
UA: ursolic acid
WED: wedelolactone
ALT: alanine aminotransferase
PAB: pseudolaric acid B
IHC: immunohistochemistry
FITC: fluorescein isothiocyanate
AST: aspartate aminotransferase
IF: immunofluorescence
IOD: integrated option density
