# Supplementary material for: Ailanthus Altissima-derived Ailanthone enhances Gastric Cancer Cell Apoptosis by Inducing the Repression of Base Excision Repair by Downregulating p23 Expression
Source: Int J Biol Sci. 2021 Jul 5;17(11):2811–25. doi: 10.7150/ijbs.60674 (PMC8326126; doi:10.7150/ijbs.60674)
Supplement: Supplementary file 1 — Supplementary figures and tables. [file ijbsv17p2811s1.pdf]

Table 1. Research status of 9 medicinal plants

Medicinal plants	Tumor type	References
Chrysin	Prostate Cancer，Ovarian Cancer	365
Hesperidin	Cholangiocyte，Oral Carcinogenesis	282
Ailanthone	Prostate Cancer，Lung Cancer	25
Indirubin	Prostate Cancer，Promyelocytic Leukemia，Glioma	157
Oridonin	Fibrosarcoma，Diffuse Large B Cell Lymphoma，Leukemia	261
Celastrol	Melanoma，Breast Cancer，Prostate Cancer	321
Ursolic acid	Colorectal Cancer，Breast Cancer，Leukemia	563
Pseudolaric Acid B	Glioma，Lung Cancer	59
Wedelolactone	Prostate Cancer，pancreatic cancer	45

Table 2 Characteristics of 93 recurrence gastric cancer patients

Characteristics			Low(51)	High(42)	*p*
Sex	Female	26	16	10	0.419
	Male	67	35	32	
Differentiation	Well	62	41	21	
	Moderate	26	7	19	0.003
	Poor	5	3	2	
pT stage	T1	22	13	9	
	T2a	13	6	7	
	T2b	28	14	14	0.870
	T3	21	13	8	
	T4	9	5	4	
pN stage	N1	58	32	26	
	N2	25	14	11	0.946
	N3	10	5	5	
pM stage	M0	84	46	38	0.851
	M1	9	5	4	

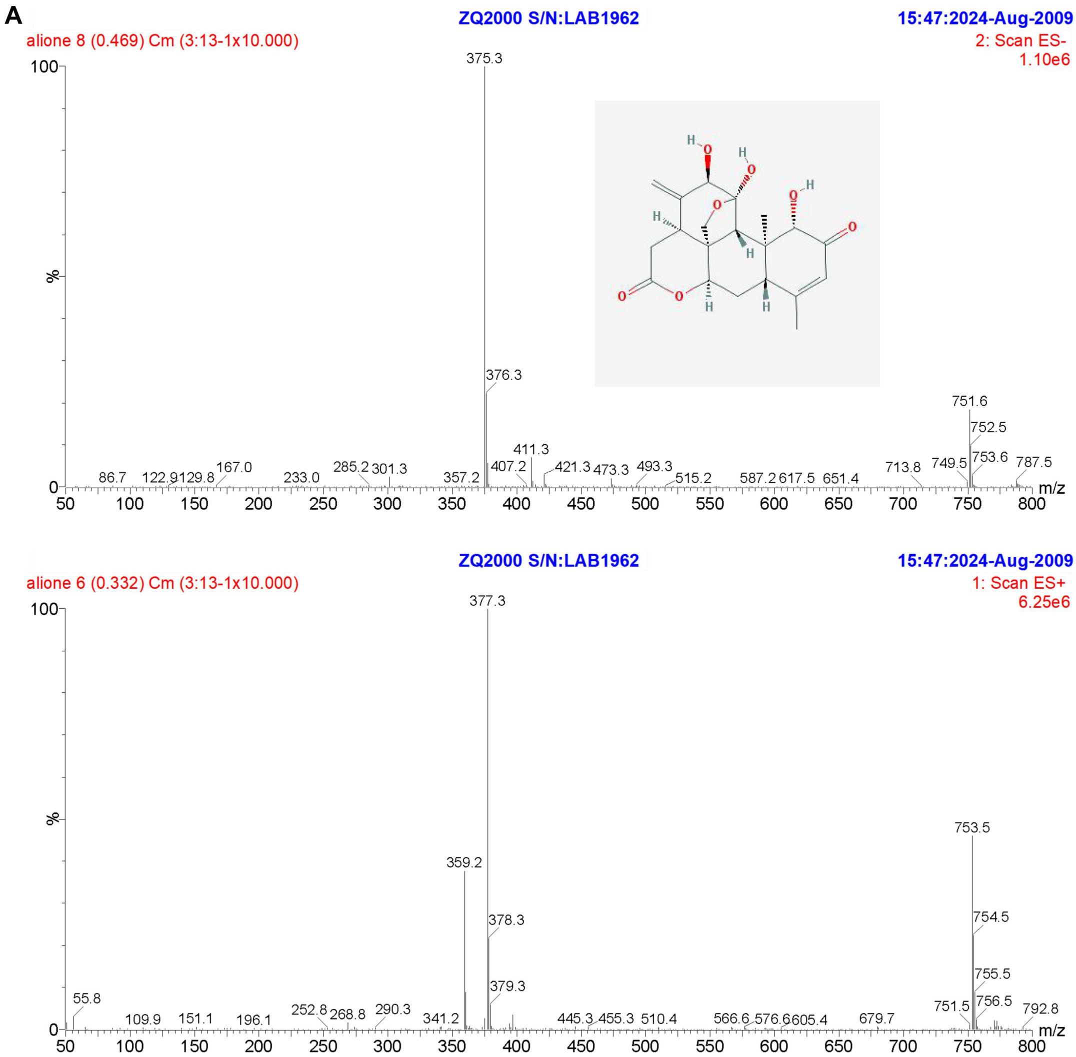

S-Fig. 1 The molecular structure and the mass spectrometry of AIL.

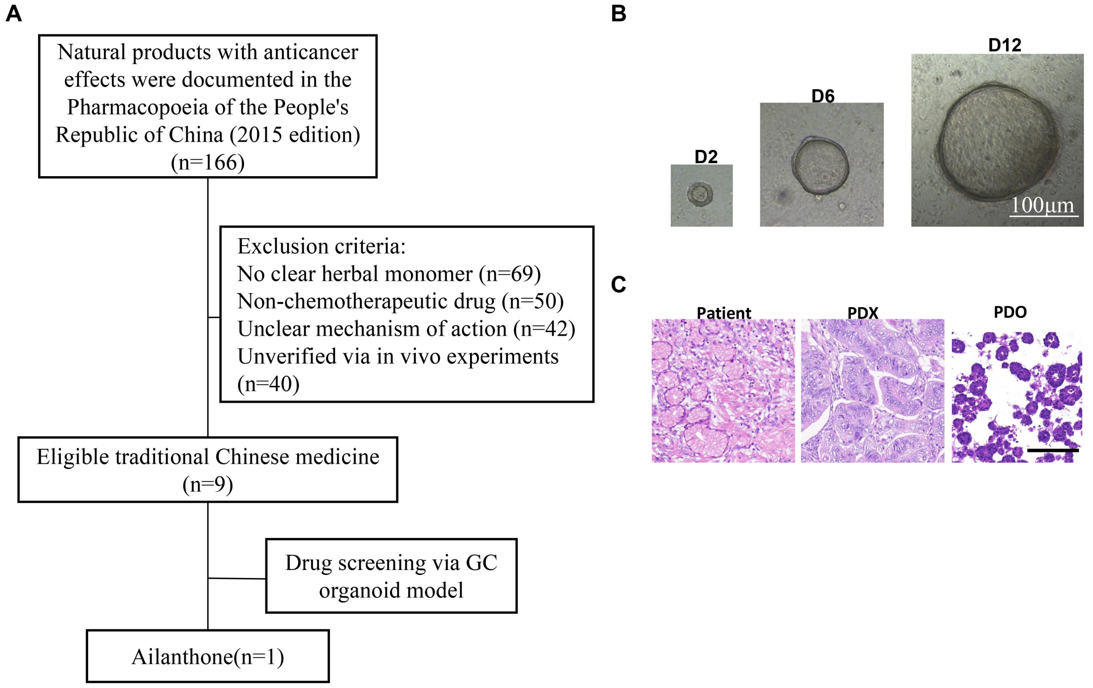

S-Fig. 2 GC organoids as a platform for drug screening. (A) Flow chart of drug screening. (B) Fresh GC tissues were collected from GC patients, and GC organoids cultured by the 3D culture method were photographed on days 2, 5, and 12 (100X). Scale bars, 200μm.(C) Representative HE-stained images of GC tissue, PDX tissue, and patient-derived organoids (PDOs) cultured from the same GC patient (200X). Scale bars, 200μm.

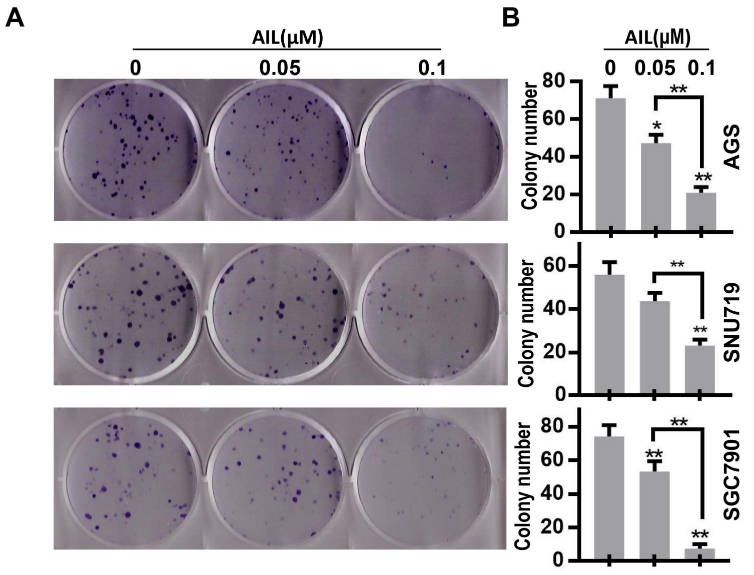

S-Fig. 3 AIL inhibits GC cell growth and colony formation. (A) AGS, SNU719 and

SGC7901 cells were treated with different concentrations of AIL (0, 0.05 and 0.1 μM) for 12 days. The colony is stained with crystal violet. (B) Data were derived from experiments conducted in triplicate in (A). The treated cells were compared with the control group, *P<0.05, **P<0.01, ***P<0.001.

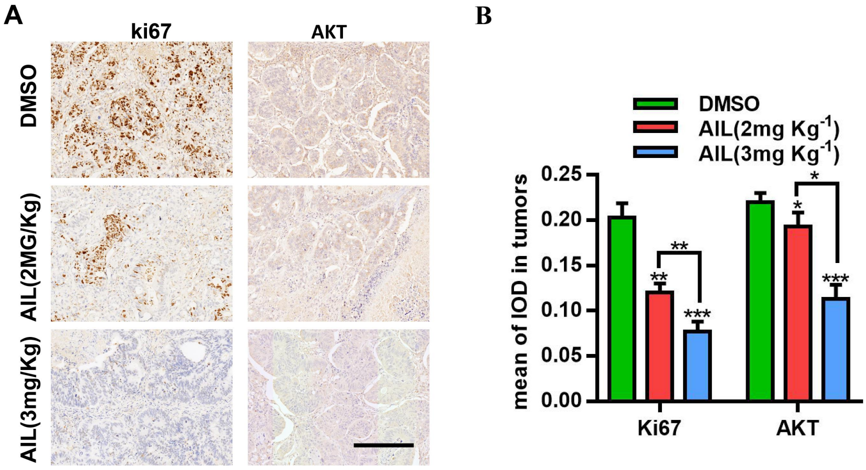

S-Fig. 4 AIL inhibits the expression of Ki67 and AKT. (A) Representative images of Ki67 and AKT in PDX tumor tissue as detected via immunohistochemistry (100X). Scale bars, 200 μm. (B) Data were derived from experiments conducted in triplicate in (C). The tumor tissues were compared with the control group, *P<0.05, **P<0.01, ***P<0.001.

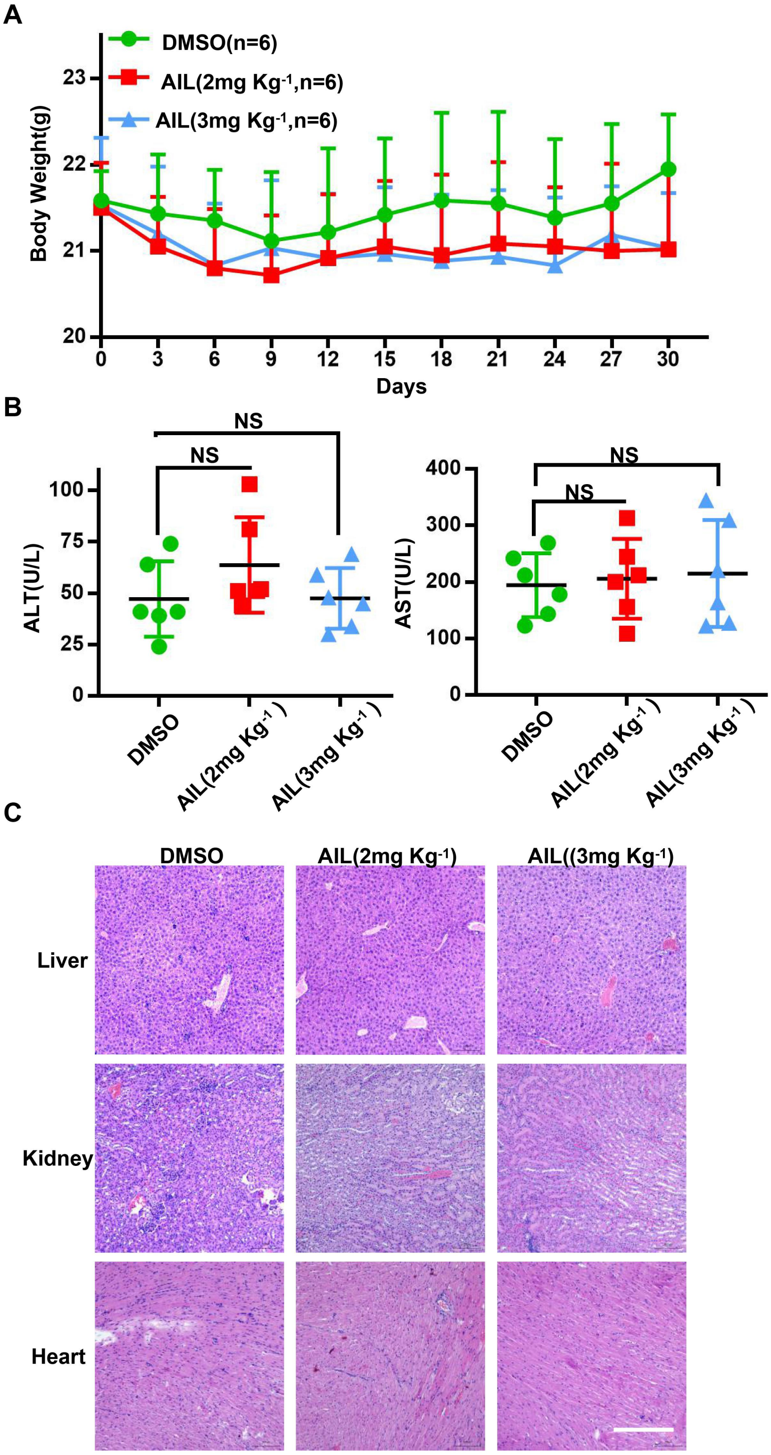

S-Fig. 5 AIL does not affect liver and kidney function. The mice were injected with 3.0 mg/kg AIL intraperitoneally every 3 days and were sacrificed on the 30th day. Serum, liver and kidney samples were collected for analysis. (A) Serum ALT and AST levels of mice. (B) Mice body mass curve. (C) HE staining was performed on the heart, liver and kidney sections and then observed under a phase-contrast microscope.

Scale bars, 200 μm.

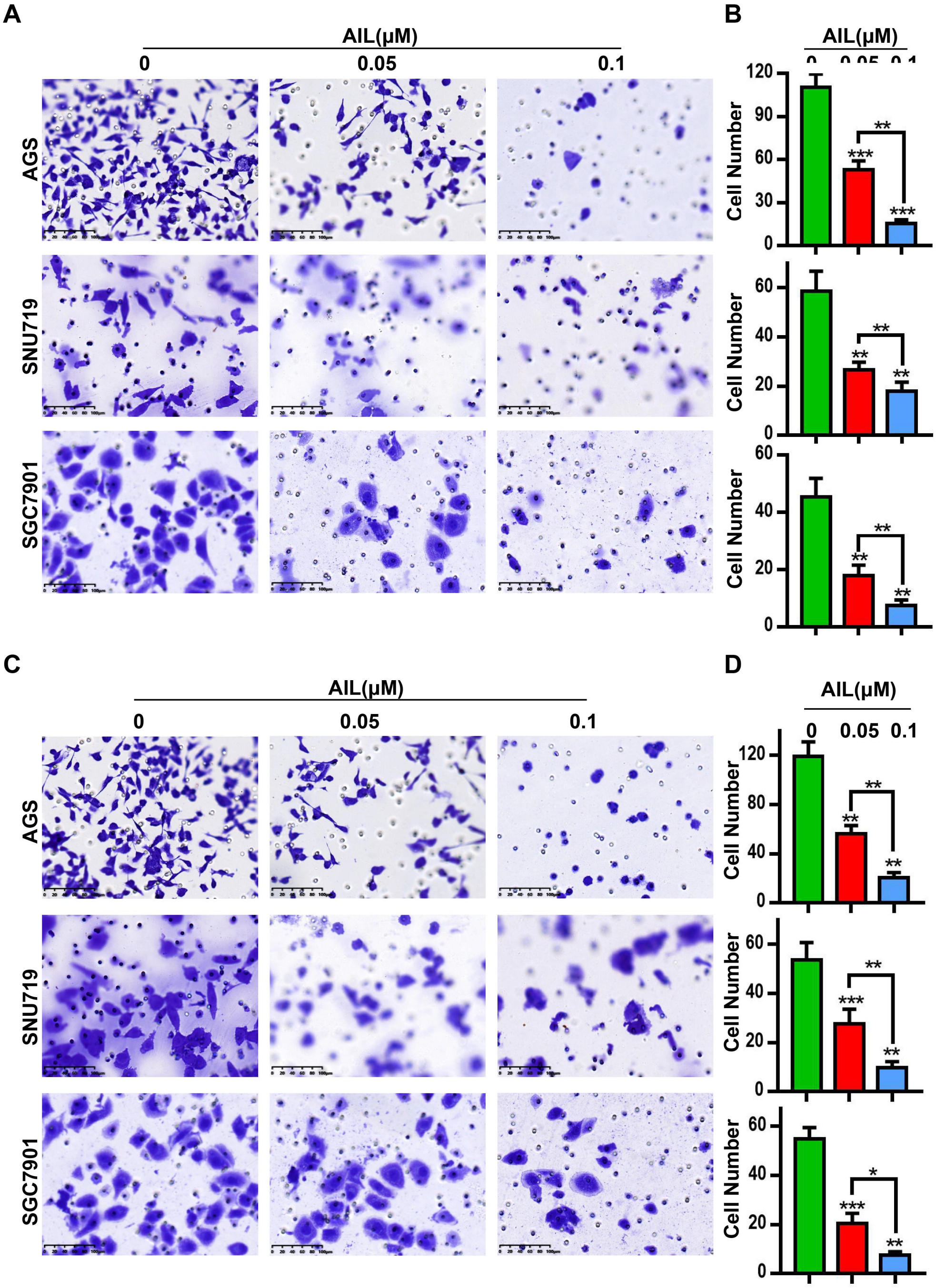

S-Fig. 6 AIL inhibits migration and invasion of GC cells. (A) Serum-free treatment of AGS, SNU719, and SGC7901 cells for 24 hours; 50,000 cells were inoculated per Transwell chamber, and then were treated with different concentrations of AIL (0, 0.05 and 0.1 μM) for 17, 24 and 42 hours. The chambers were then fixed with methanol, stained with crystal violet, mounted and scanned. (B) Data were derived from experiments conducted in triplicate in (A). (C) Serum-free treatment of AGS,

SNU719, and SGC7901 cells for 24 hours. A 100 μl Matrigel mixture (Matrigel: water = 1:8) was spread on the base of the Transwell chamber, and 100,000 cells were inoculated per well. After treatment with different concentrations of AIL (0, 0.05 and 0.1 μM) for 17, 24, and 42 hours, cells were fixed with methanol, stained with crystal violet, mounted and scanned. (D) Data were derived from experiments conducted in triplicate in (C). The treated cells were compared with the control group, *P<0.05, **P<0.01, ***P<0.001.

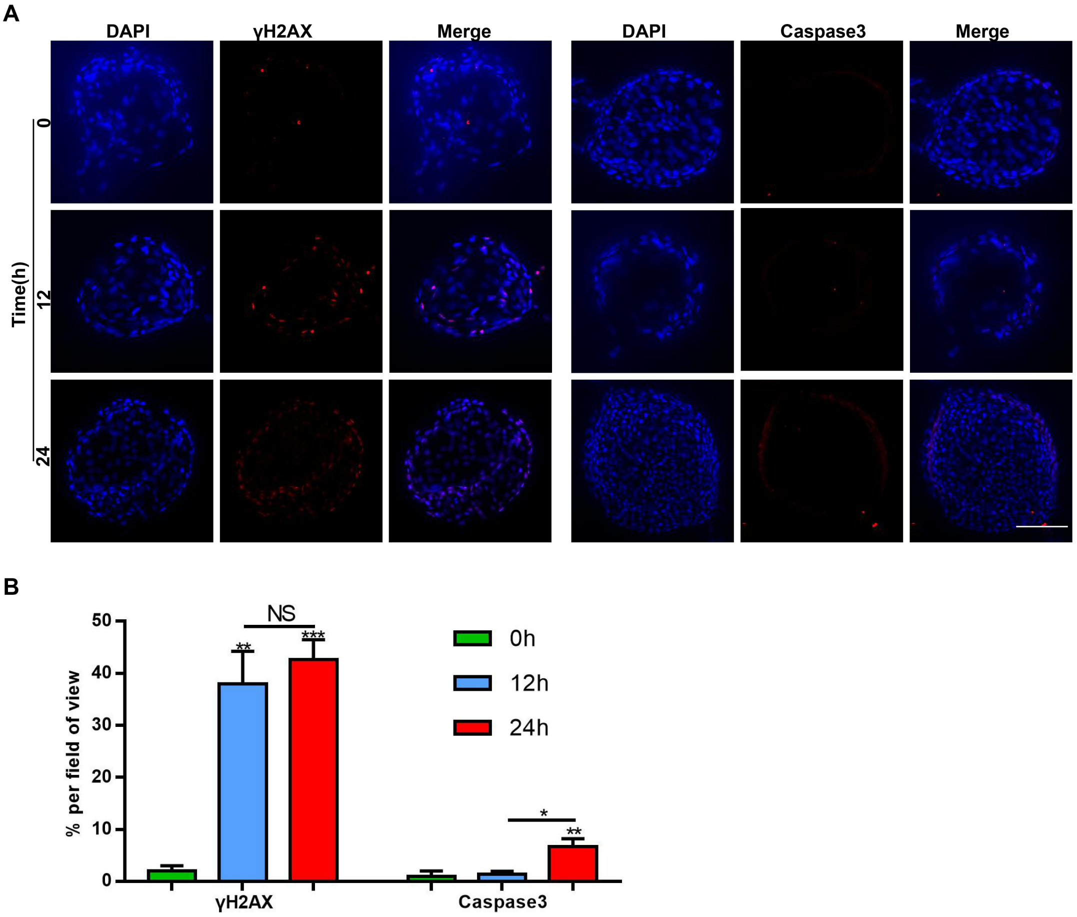

S-Fig. 7 AIL induces DNA damage without apoptosis in GC organoids at an early stage. GC organoids were embedded and sectioned after treatment with DMSO (control group) and AIL (1 μM) for 12 and 24 hours. An immunofluorescence assay was performed to analyze the expression of γH2AX and Caspase3. Scale bars, 100 μm.

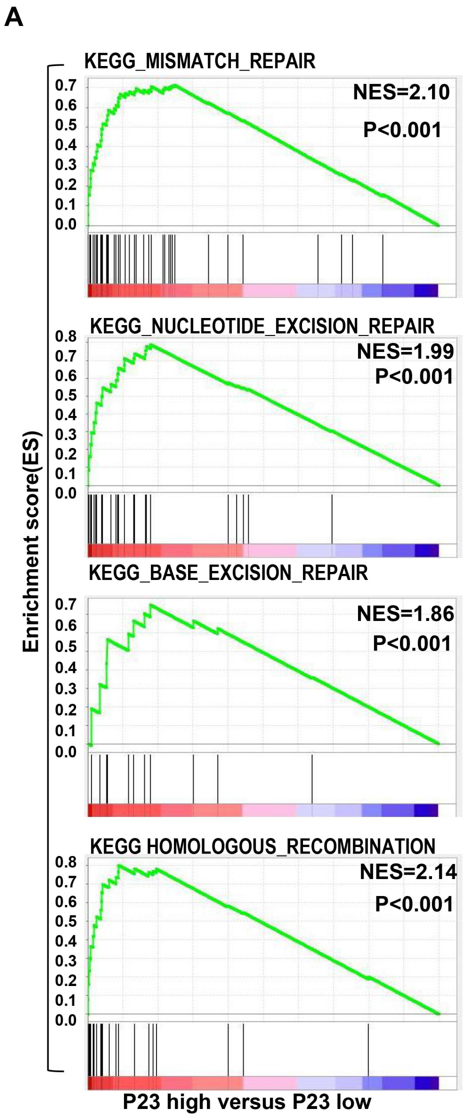

S-Fig. 8 P23 participates in DNA repair. Bioinformatics methods revealing the relationship between P23 and DNA repair-related pathways.

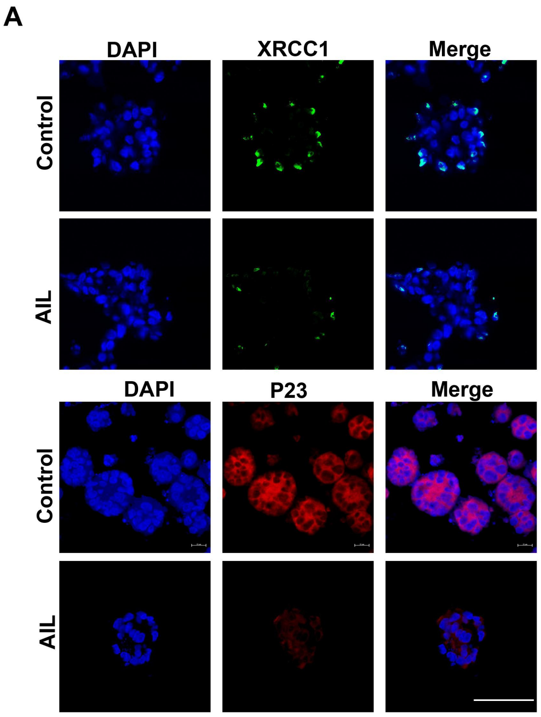

S-**Fig. 9 AIL inhibits P23 and XRCC1 in GC organoids.** Representative images of P23 and XRCC1 in GC organoids as detected via IF (400X). Scale bars, 100 μm.

B

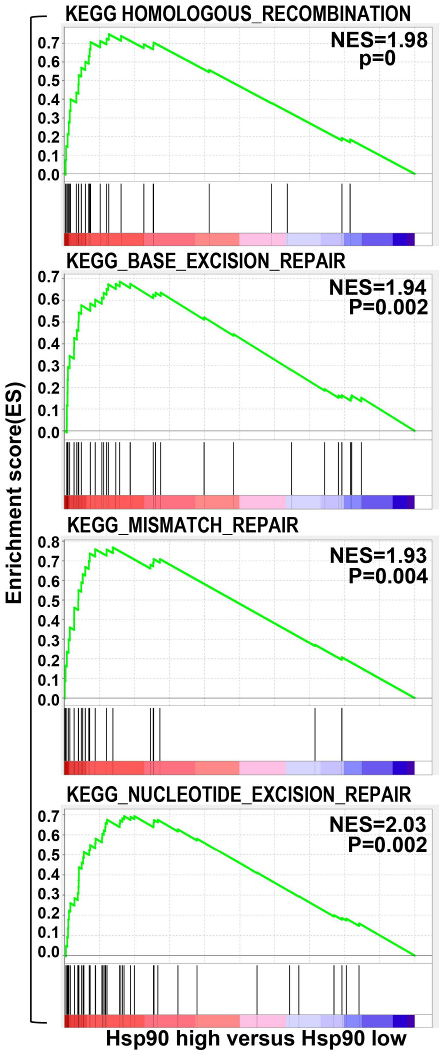

S-Fig. 10 HSP90 promotes DNA repair. Bioinformatics methods revealing the relationship between HSP90 and DNA repair-related pathways.

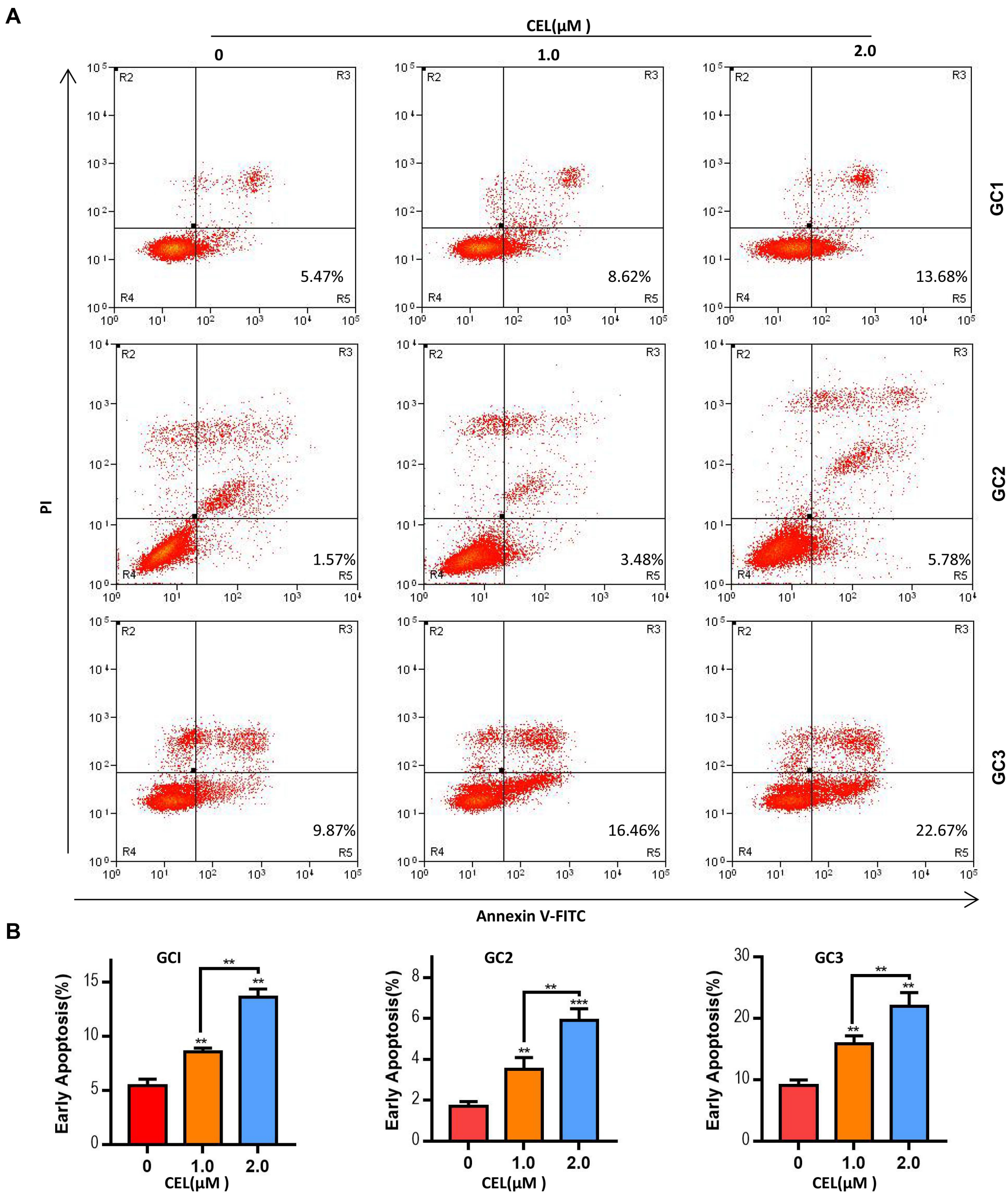

S-Fig. 11 CEL induces apoptosis in GC organoids. (A) Three GC organoids were treated with DMSO (control group) and specified concentrations of CEL for 48 hours, followed by digestion into single cells and analysis of apoptosis through flow cytometry analysis with FITC-Annexin V and PI staining. (B) Data were derived from experiments conducted in triplicate in (A).
